# Missed Proximal Tracheoesophageal Fistula (TEF) in a Neonate with Type D Esophageal Atresia

**DOI:** 10.1055/a-2227-6389

**Published:** 2024-01-10

**Authors:** Julia E. Menso, Maud A. Reijntjes, Matthijs W. Oomen, Rico N.P.M. Rinkel, Suzanne W.J. Terheggen-Lagro, Ramon R. Gorter

**Affiliations:** 1 Department of Pediatric Surgery, Emma's Children's Hospital Amsterdam UMC, location University of Amsterdam, Meibergdreef 9, 1105 AZ Amsterdam, The Netherlands; 2 Amsterdam Gastroenterology and Metabolism Research Institute, 1105 AZ Amsterdam, The Netherlands; 3 Amsterdam Reproduction and Development Research Institute, 1105 AZ Amsterdam, The Netherlands; 4 Department of Otolaryngology, Emma's Children's Hospital Amsterdam UMC, location University of Amsterdam, Meibergdreef 9, 1105 AZ Amsterdam, The Netherlands; 5 Department of Pediatric Pulmonology, Emma's Children's Hospital Amsterdam UMC, location University of Amsterdam, Meibergdreef 9, 1105 AZ Amsterdam, The Netherlands

**Keywords:** esophageal atresia, type D, tracheoesophageal fistula, diagnosis

## Abstract

We present the case of a patient with the rare type D esophageal atresia (EA), diagnosed after correction of an EA initially diagnosed as type C. Routine postoperative contrast esophagogram showed a missed proximal tracheoesophageal fistula. This case report illustrates the potential difficulties to diagnose type D EA.

## Introduction

Esophageal atresia (EA) is a rare congenital malformation with a reported incidence up to 1 in 3,000 live births worldwide, and can be classified according to the Gross classification. 1 This classification anatomically defines EA as types A to E, based on the relation between the esophageal pouches and the trachea. Type D is the rarest EA with double tracheoesophageal fistula (TEF) from the proximal and distal esophageal pouch, which occurs in 1 to 3% of EA cases. 2 3 4 Regular diagnostic workup of EA includes a chest X-ray with curling nasogastric tube (NGT) in the proximal pouch. In the presence of air in the stomach and/or the bowel, the diagnosis of EA with a distal TEF is confirmed and patients will be scheduled for surgical correction. 4 One must always be aware of type D EA in which both a distal and a proximal fistula are present, the latter being hard to identify. Both preoperative esophagoscopy and bronchoscopy may identify a proximal fistula in children undergoing EA repair. 5 6 7 We present the case of an infant with the type D EA, diagnosed after correction of an EA initially suspected type C.

## Case Report

**Fig. 1:**
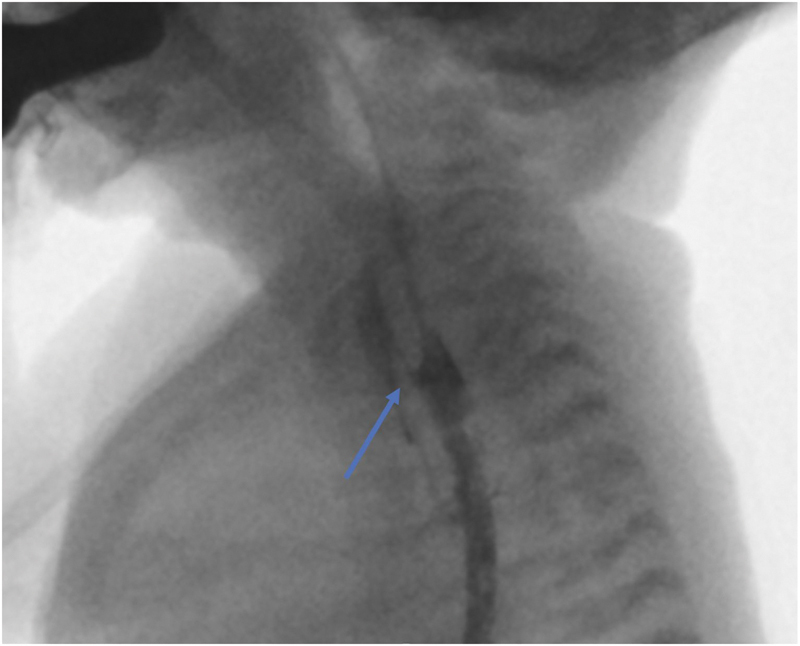
Fluoroscopy using iodine contrast (Iohexol). The lateral view did not reveal a fistula. Rolling the patient toward the abdomen revealed a proximal tracheoesophageal fistula at the anastomotic level (pointed out by the arrow in the figure).

**Fig. 2:**
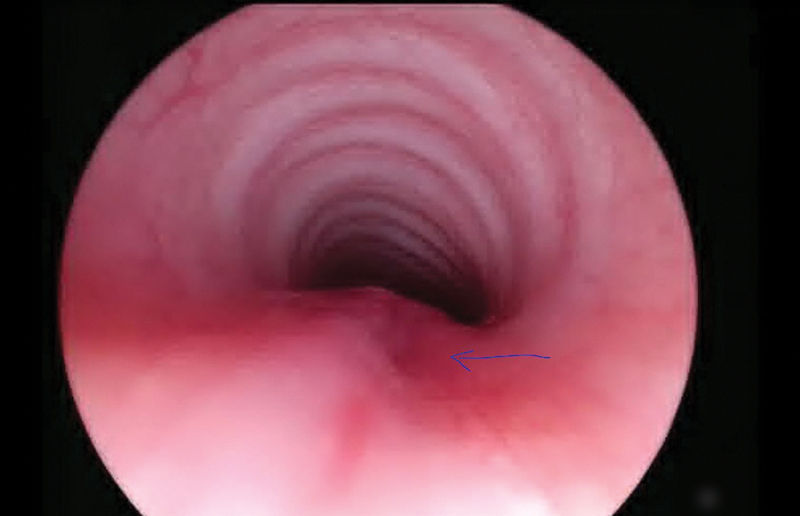
Preoperative bronchoscopy prior to the second surgical procedure showed only a small dimple in the proximal trachea (pointed out by the arrow), without a clear lumen of a fistula.

A preterm male neonate with a gestational age of 34 weeks and birth weight of 1,586 g was admitted at the neonatal intensive care unit at the Amsterdam University Medical Center for respiratory support. During pregnancy, no medication was used. The noninvasive prenatal test showed no abnormalities. The pregnancy was ultimately complicated by vasa praevia, intrauterine growth restriction, and impending preterm birth. Cardiac dextroposition and a ventricular septal defect (VSD) were diagnosed antenatal by ultrasonography. Postnatally, the patient experienced clinical symptoms compatible with EA. Therefore, a Replogle tube was placed. On chest X-ray, the Replogle tube stagnated at the second costal bone and fourth thoracic vertebra and air was noted in the stomach and bowel. Diagnosis of EA with distal TEF was confirmed. Preoperatively, echocardiography showed a dextroposition of the heart with a surgically irrelevant VSD. A preoperative diagnostic rigid laryngotracheobronchoscopy was performed, according to which the otorhinolaryngologist diagnosed severe tracheomalacia and identified only one prominent distal tracheoesophageal fistula. Besides the tracheal, esophageal, and cardiac abnormalities, the vertebral, anal, renal, and limb (VACTERL) screening revealed a vertebral segmentation disorder without a tethered cord. EA correction with right-sided thoracotomy was performed on the third day postpartum. The confirmed distal fistula was ligated, the proximal esophageal pouch was mobilized toward the thoracic inlet as much as possible, and considered to be safe. Due to the short length of the proximal pouch, internal traction was performed with two PDS 4–0 traction sutures, whereafter the proximal and distal esophageal pouches were approximated, and an anastomosis (including placement of a transanastomotic NGT) was created. Due to tension on the anastomosis, a thoracic drain was left in situ to monitor postoperative anastomotic leakage and it was decided that a contrast esophagogram should be performed on day 7 to exclude any leakage. No intraoperative complications occurred. The first day postoperatively, enteral feeding via the transanastomotic NGT was initiated and subsequently increased to full enteral nutrition. The thoracic drain showed no production. At the seventh day postoperatively, a contrast esophagogram demonstrated a potential recurrent or iatrogenic fistula without prominent anastomotic leakage or stenosis. A repetitive esophagogram was undertaken for more clarification and showed an evident tracheoesophageal connection proximal to the anastomosis (thoracic inlet level), suggesting a missed proximal fistula (see Fig. 1 ). During this second esophagogram, the patient experienced a respiratory incident. The patient was rescheduled for surgical correction on the 11th day after birth. Preoperative flexible and rigid bronchoscopies showed only a small dimple in the proximal trachea 2 cm caudal to the cricoid, without a clear lumen of a fistula (see Fig. 2 ). A trial to introduce a Fogarty catheter as a guidewire was not successful. Therefore, a rigid esophagoscopy was performed, which identified the proximal fistula with an eminent open lumen. Due to the proximal location of the fistula, a cervical approach was used to identify and ligate the proximal TEF. A flap of the sternothyroid muscle was used to prevent recurrence. Seven days postoperatively, a chest X-ray with contrast showed no anastomotic leakage nor TEF, whereafter enteral feeding via the transanastomotic NGT was increased to full enteral nutrition. As feeding via the NGT was tolerated properly, oral intake was initiated, and tube feeding was subsequently reduced. At the age of 2 months, the patient underwent aortopexy for his tracheomalacia. Three months after repair of the proximal fistula, complete oral intake was achieved.

## Discussion

Diagnosis of a second proximal fistula in type D EA can be challenging as chest X-ray with contrast or bronchoscopy lacks sensitivity. 8 We presented the case of a patient with EA in which the proximal fistula was not identified by initial routine bronchoscopy before primary reconstruction. The diagnosis was made on a contrast study performed on the seventh day after surgical correction, initially done to exclude anastomotic leakage. The incidence of missed proximal fistula is reported to be 4 to 6%. 9 10 The clinical diagnosis of recurrent or missed fistula is challenging as most patients also suffer from gastroesophageal reflux or tracheomalacia, symptoms compatible with TEF (e.g., coughing and respiratory incidents). 8 10

The diagnostic laryngotracheobronchoscopy prior to the first surgical procedure was performed by an ear, nose, and throat (ENT) staff member who is experienced in tracheobronchoscopies and EA. It did not show abnormalities in the proximal trachea, and esophagoscopy was not performed. Hence, treatment for type C EA was initiated. Respiratory support during the diagnostic laryngotracheobronchoscopy might have occluded the proximal fistula as the endotracheal tube that was present during the procedure to close the distal fistula will have blocked the proximal fistula, thus letting potential air leakage after closure of the distal fistula go unnoticed. With an ambiguous finding in the diagnostic laryngotracheobronchoscopy, additional esophagoscopy proved its additional value in confirming the presence of a missed proximal fistula of type D EA. The esophageal entrance of the fistula was larger than the opening of the fistula on the tracheal side. This funnel-shaped presentation of the fistula is most likely the explanation that on the first endoscopy the fistula was missed. Repair of a proximal fistula is mostly recommended via a cervical approach, although thoracoscopic approaches have also been reported. 8 11

## Conclusion

This case report illustrates the potential difficulties to diagnose type D EA. In our case, it was diagnosed coincidentally prior to developing clinical symptoms suggestive of a recurrent or missed proximal fistula. In case there is a suspicion of a recurrent fistula, additional diagnostics (i.e., contrast esophagogram, bronchoscopy and esophagoscopy) should be undertaken to also consider a missed proximal fistula of type D EA.

## Notes

**Erratum:** The article has been corrected as per Erratum published on February 16, 2024 (DOI: 10.1055/s-0044-1779612).
