# Erratum: Missed Proximal Tracheoesophageal Fistula (TEF) in a Neonate with Type D Esophageal Atresia

**DOI:** 10.1055/s-0044-1779612

**Published:** 2024-02-16

**Authors:** Julia E. Menso, Maud A. Reijntjes, Matthijs W. Oomen, Rico N.P.M. Rinkel, Suzanne W.J. Terheggen-Lagro, Ramon R. Gorter

**Affiliations:** 1 Department of Pediatric Surgery, Emma's Children's Hospital Amsterdam UMC, location University of Amsterdam, Meibergdreef 9, 1105 AZ Amsterdam, The Netherlands; 2 Amsterdam Gastroenterology and Metabolism Research Institute, 1105 AZ Amsterdam, The Netherlands; 3 Amsterdam Reproduction and Development Research Institute, 1105 AZ Amsterdam, The Netherlands; 4 Department of Otolaryngology, Emma's Children's Hospital Amsterdam UMC, location University of Amsterdam, Meibergdreef 9, 1105 AZ Amsterdam, The Netherlands; 5 Department of Pediatric Pulmonology, Emma's Children's Hospital Amsterdam UMC, location University of Amsterdam, Meibergdreef 9, 1105 AZ Amsterdam, The Netherlands

In the above article published on January 10, 2024 (DOI: 10.1055/a-2227-6389 ), the authors have brought to the Publisher's attention that the affilations were tagged incorrectly. The correct affiliations appear as above.

